# Supplementary material for: Activation of the 20S proteasome core particle prevents cell death induced by oxygen- and glucose deprivation in cultured cortical neurons
Source: Apoptosis. 2025 Mar 17;30(5-6):1372–90. doi: 10.1007/s10495-025-02097-x (PMC12167278; doi:10.1007/s10495-025-02097-x)
Supplement: Supplementary file 1 — Supplementary Material 1 [file 10495_2025_2097_MOESM1_ESM.docx]

**Supplementary material**

Anti-Rpt6 (referred to Figure 1E) Anti-tubulin WB (referred to Figure 1E)


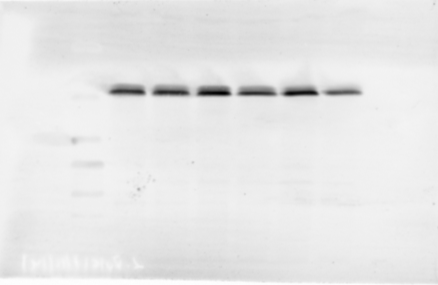

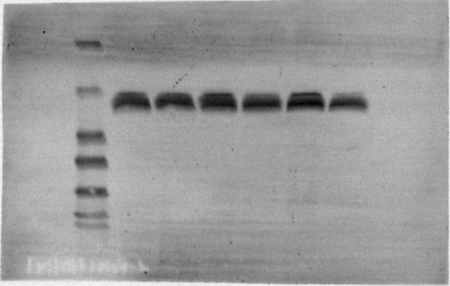


Anti-Psma2 (referred to Figure 1F) Anti-tubulin WB (referred to Figure 1F)


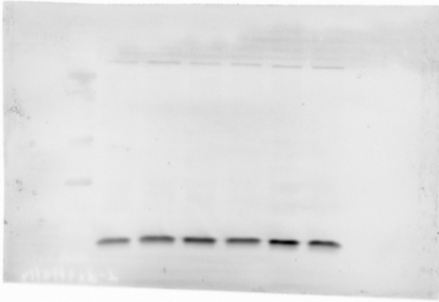

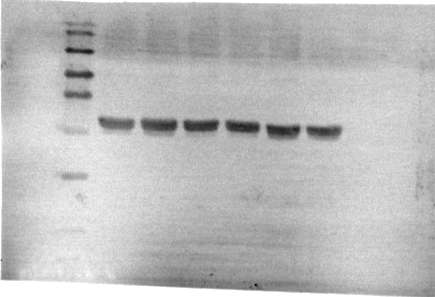


**Figure 1 – Supplementary material**

Anti-spectrin WB (referred to Figure 5A) Anti-tubulin WB (referred to Figure 5A)


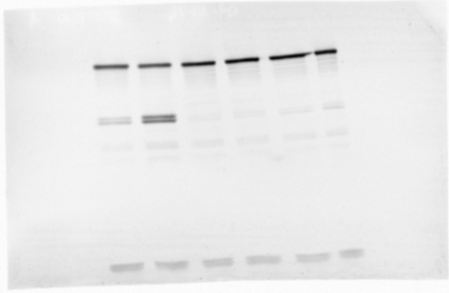

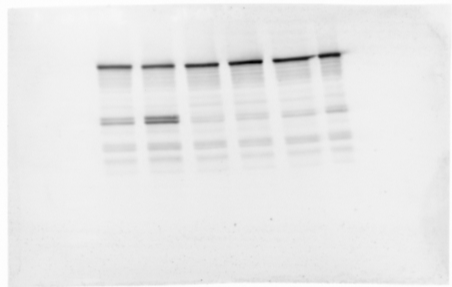


Anti-Rpn10 WB referred to Figure 5B) Anti-tubulin WB (referred to Figure 5B)


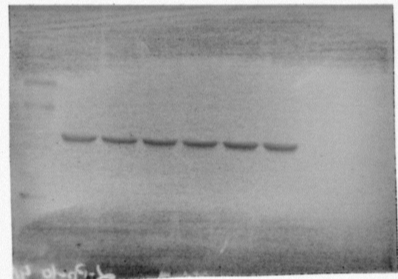

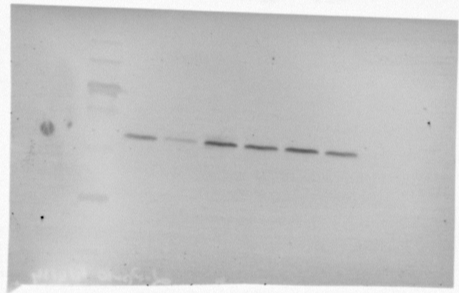


Anti-Rpt3 WB (referred to Figure 5C) Anti-tubulin WB (referred to Figure 5C)


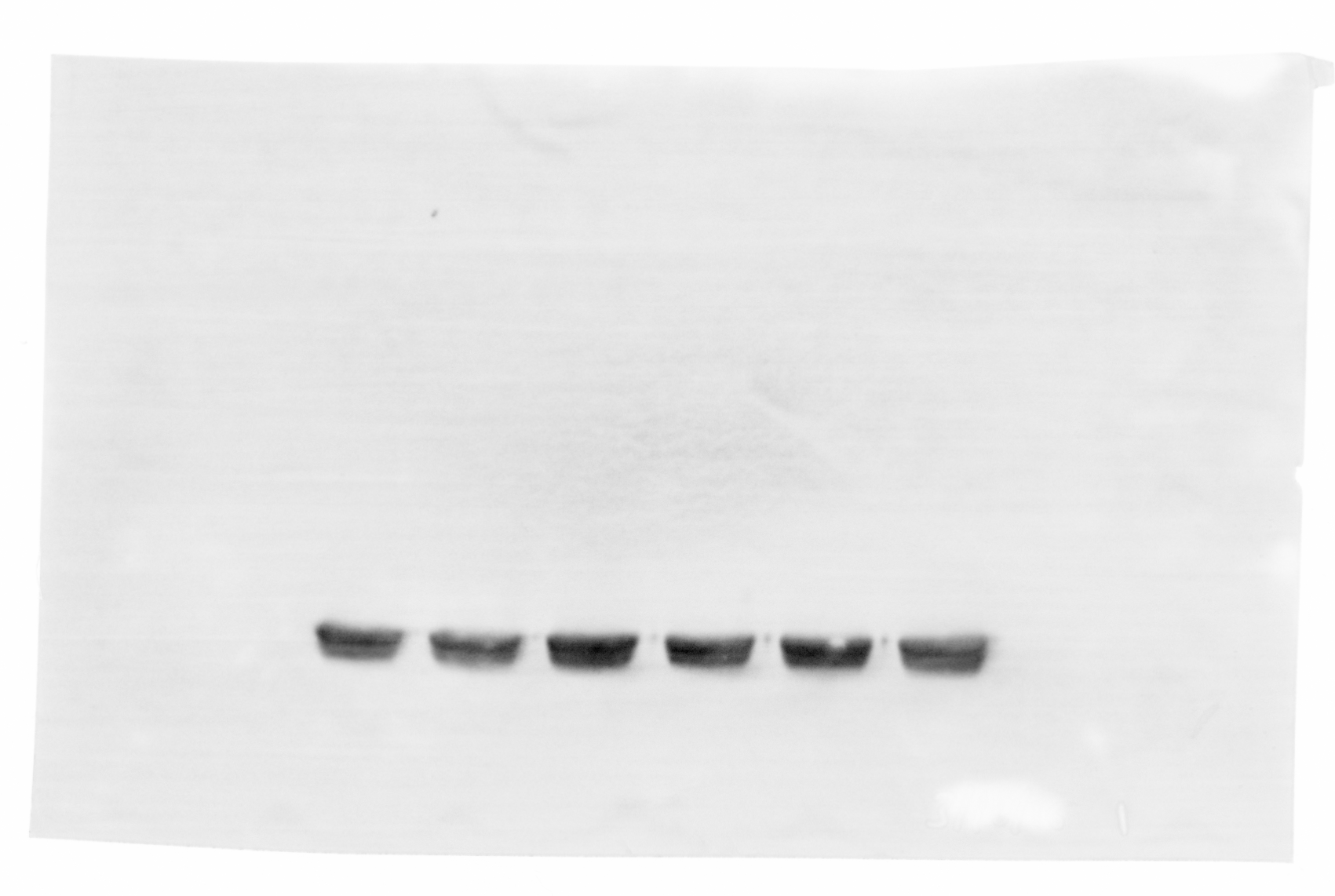

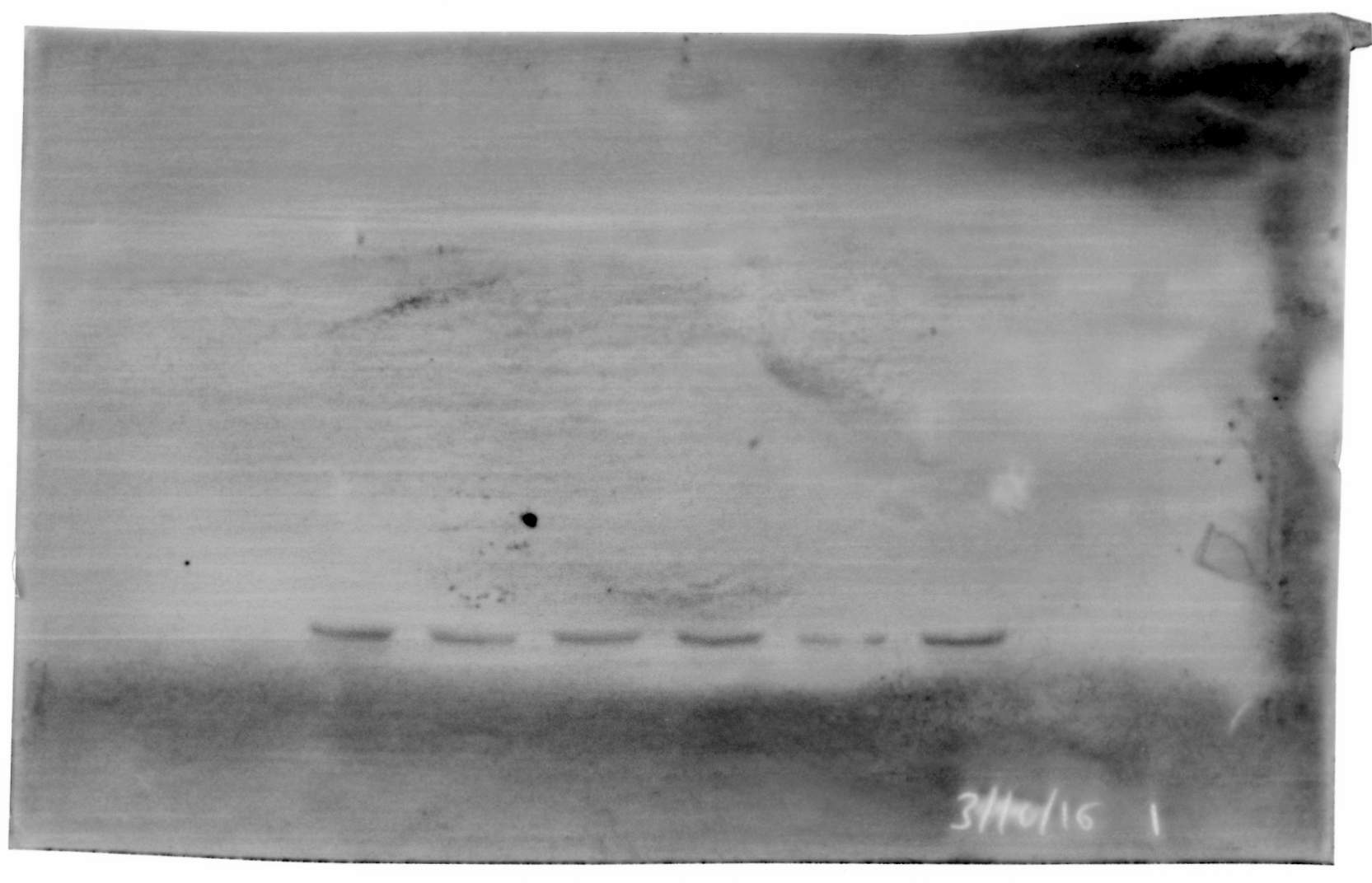


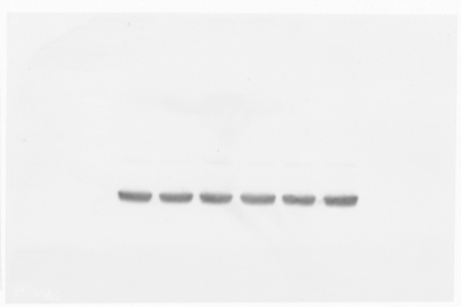
Anti-Rpn6 WB (referred to Figure 5D) Anti-tubulin WB (referred to Figure 5D)


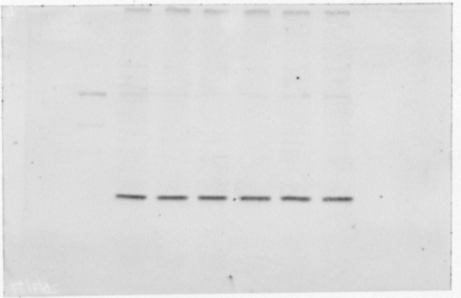


**Figure 5 – Supplementary material**

Anti-Spectrin WB (referred to Figure 6D) Anti-tubulin WB (referred to Figure 6D)


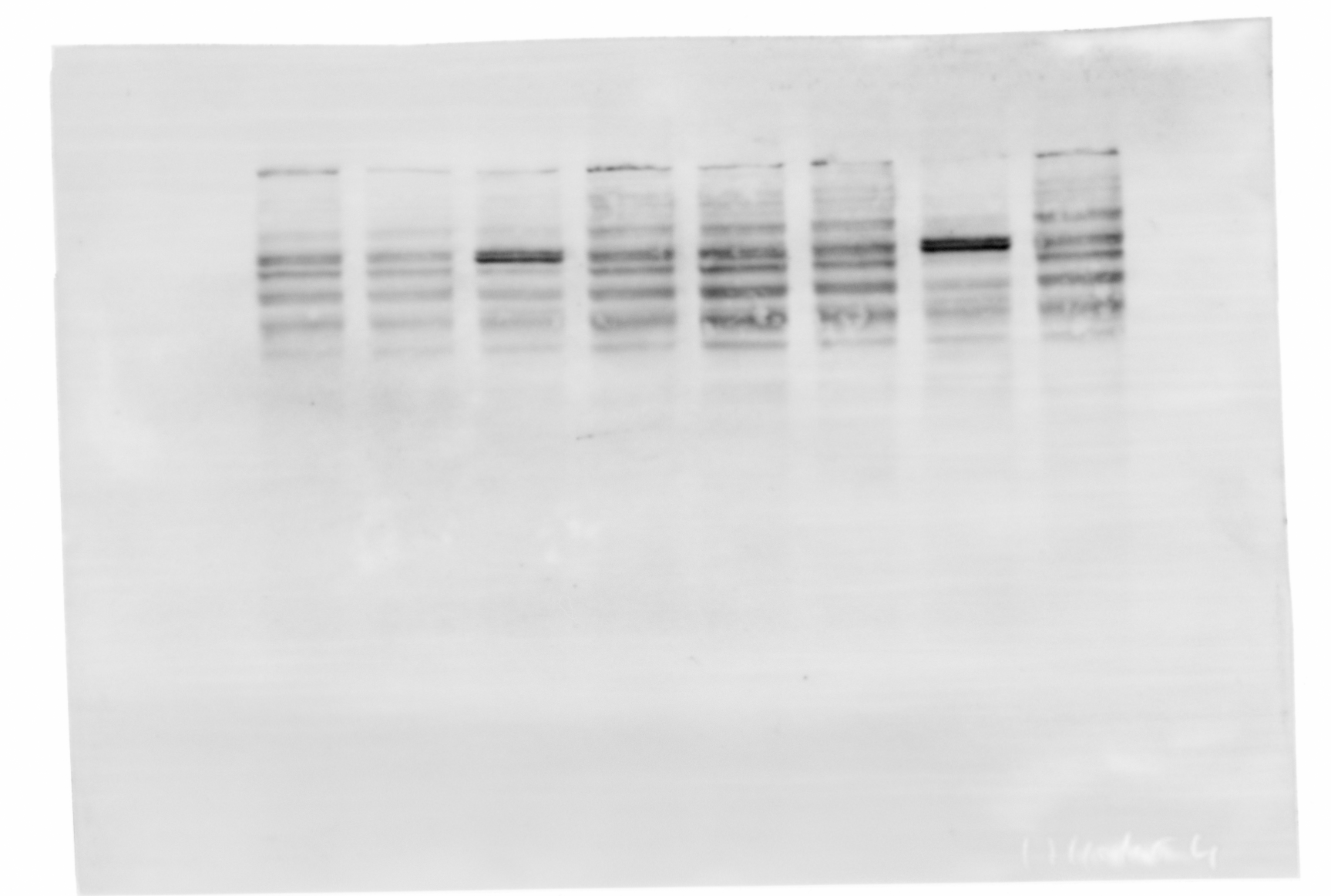

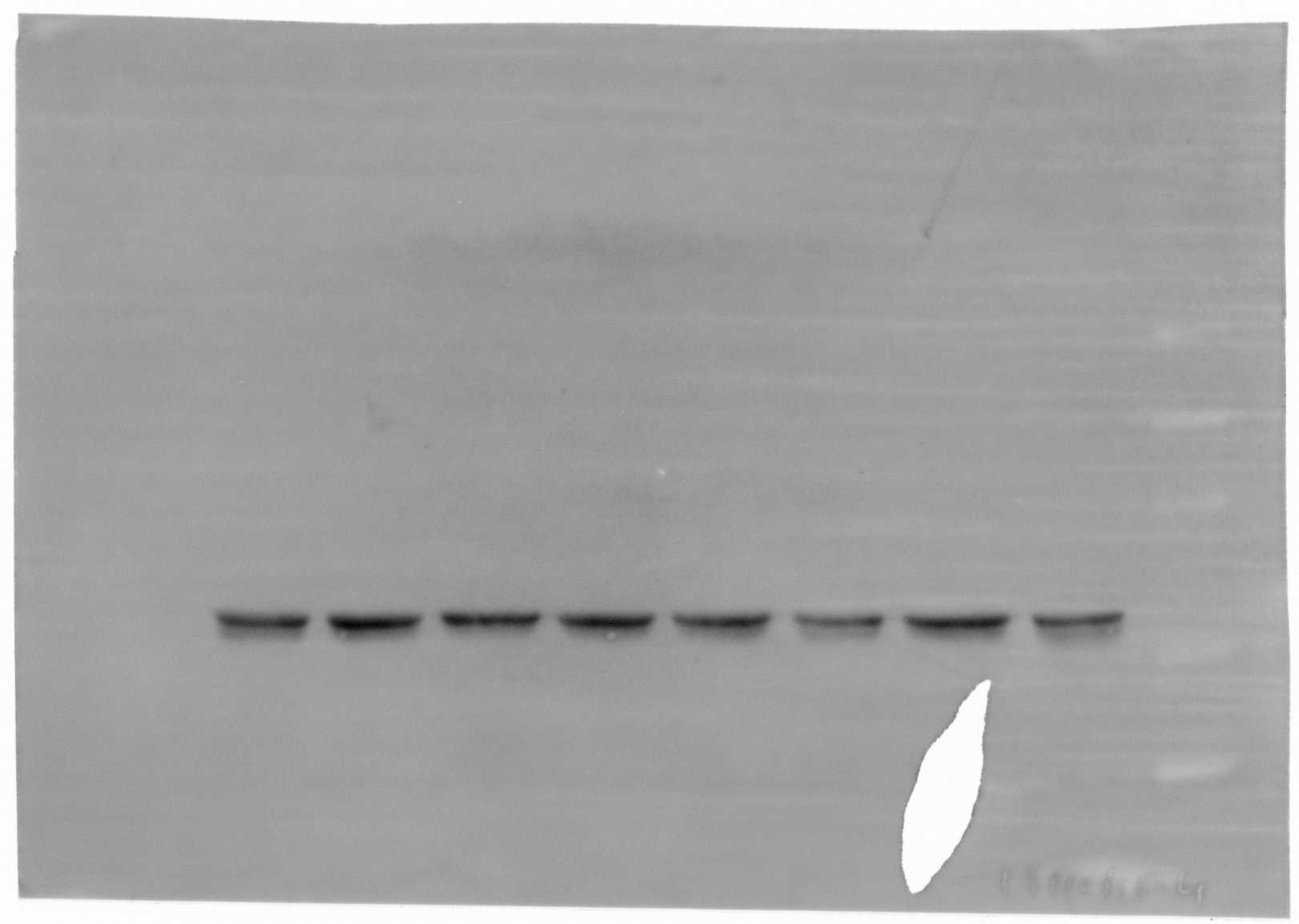


**Figure 6 – Supplementary material**

Anti-Spectrin WB (referred to Figure 7C) Anti-tubulin WB (referred to Figure 7C)


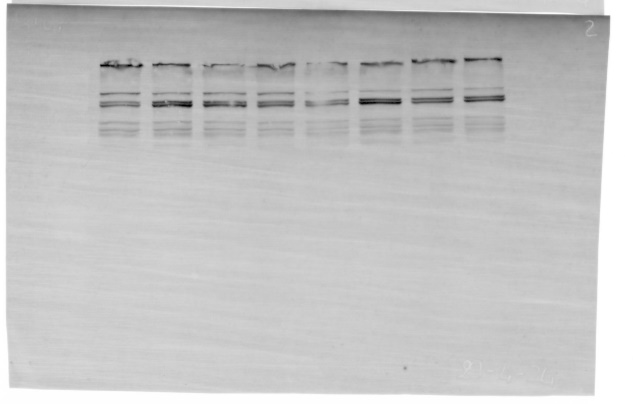

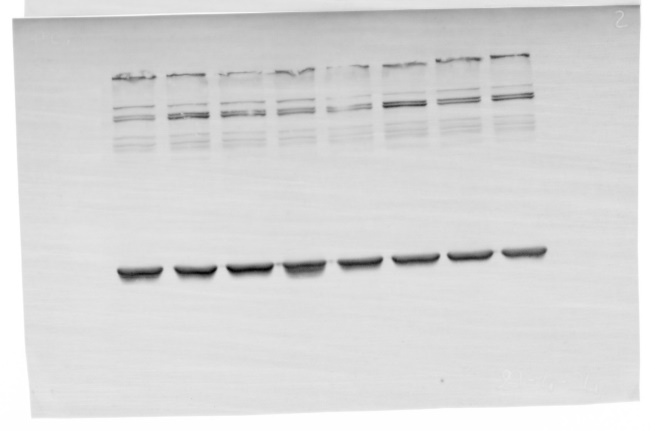


**Figure 7 – Supplementary material**
